# Maternal high-fat diet-induced programing of gut taste receptor and inflammatory gene expression in rat offspring is ameliorated by CLA supplementation

**DOI:** 10.14814/phy2.12588

**Published:** 2015-10-22

**Authors:** Clare M Reynolds, Stephanie A Segovia, Xiaoyuan D Zhang, Clint Gray, Mark H Vickers

**Affiliations:** Liggins Institute and Gravida: National Centre for Growth and Development, University of AucklandAuckland, New Zealand

**Keywords:** Developmental programing, gut hormones, inflammation, maternal high fat, taste receptors

## Abstract

Consumption of a high-fat (HF) diet during pregnancy and lactation influences later life predisposition to obesity and cardiometabolic disease in offspring. The mechanisms underlying this phenomenon remain poorly defined, but one potential target that has received scant attention and is likely pivotal to disease progression is that of the gut. The present study examined the effects of maternal supplementation with the anti-inflammatory lipid, conjugated linoleic acid (CLA), on offspring metabolic profile and gut expression of taste receptors and inflammatory markers. We speculate that preventing high-fat diet-induced metainflammation improved maternal metabolic parameters conferring beneficial effects on adult offspring. Sprague Dawley rats were randomly assigned to a purified control diet (CD; 10% kcal from fat), CD with CLA (CLA; 10% kcal from fat, 1% CLA), HF (45% kcal from fat) or HF with CLA (HFCLA; 45% kcal from fat, 1% CLA) throughout gestation and lactation. Plasma/tissues were taken at day 24 and RT-PCR was carried out on gut sections. Offspring from HF mothers were significantly heavier at weaning with impaired insulin sensitivity compared to controls. This was associated with increased plasma IL-1*β* and TNF*α* concentrations. Gut Tas1R1, IL-1*β*, TNF*α*, and NLRP3 expression was increased and Tas1R3 expression was decreased in male offspring from HF mothers and was normalized by maternal CLA supplementation. Tas1R1 expression was increased while PYY and IL-10 decreased in female offspring of HF mothers. These results suggest that maternal consumption of a HF diet during critical developmental windows influences offspring predisposition to obesity and metabolic dysregulation. This may be associated with dysregulation of taste receptor, incretin, and inflammatory gene expression in the gut.

## Introduction

Obesity is fast becoming the most common chronic health problem worldwide. It is pivotal in the development of insulin resistance (IR) and subsequent cardiometabolic disease. There are numerous influences which contribute increased obesity rates with genetics, sedentary lifestyle, and availability of inexpensive calorie dense food all representing key risk factors. In modern Western societies, the proportion of women of child-bearing age presenting as overweight or obese has also increased (Chu et al. 2007) and represents about two-thirds of women in the United States (Hillemeier et al. 2011). In addition to increased risk of pregnancy complications such as pre-eclampsia and gestational diabetes, maternal obesity and increased caloric intake during pregnancy can increase the risk for obesity and associated metabolic dysfunction in offspring, thereby perpetuating the cycle of obesity into the next generation (Taylor and Poston 2007; Alfaradhi and Ozanne 2011).

There is significant evidence that excess maternal caloric intake results in dysregulation of adipose, renal, and pancreatic tissues in offspring culminating in increased risk for obesity and metabolic disease (McMillen and Robinson 2005). Despite the clear role of the gastrointestinal system in development of obesity and metabolic complications, the contribution of the gut in the context of developmental programing, has been somewhat neglected. Recent studies have shown the importance of taste receptors in the gut, particularly in regard to glucose sensing and uptake. Activation of these receptors stimulates production of bioactive peptides such as peptide YY (PYY), glucagon-like peptide (GLP)-1, and ghrelin which play an important role in satiety and metabolic processes (Calvo and Egan 2015). Furthermore, recent studies have indicated that patients with type-2 diabetes have dysregulated gut taste sensing systems resulting in an increased risk of postprandial hyperglycemia (Young et al. 2013).

Animal models have demonstrated that the effects of an adverse maternal environment on later offspring metabolic and cardiovascular risk can be ameliorated via nutritional and pharmacologic interventions during the early period of developmental plasticity (Vickers and Sloboda 2015). As obesity is associated with low-grade chronic inflammation and progression to metabolic disease (Wieser et al. 2013), we sought to design an intervention strategy which neutralized obesity-induced metabolic inflammation in the mother through supplementation with a dietary anti-inflammatory nutrient. Conjugated linoleic acid (CLA) is lipid commonly found in beef and dairy produce. There are currently 28 known isomers with the c9,t11-CLA and t10-c12-CLA isomers responsible for the observed anti-inflammatory and antiobesity effects (Moloney et al. 2014; Viladomiu et al. 2007). Furthermore, there is evidence that CLA supplementation promotes gut health (Reynolds et al. 2014; Bassaganya-Riera et al. 2010). We hypothesize that supplementation with CLA dampens maternal metabolic stress caused by high-fat diet consumption which in turn prevents developmental programing of metabolic dysfunction in offspring. We therefore aimed to determine whether CLA supplementation during pregnancy could impact maternal inflammatory profiles and subsequent metabolic health of male and female offspring at weaning, particularly in relation to gut health.

## Materials and Methods

### Animal model

An established model of maternal high-fat diet-induced obesity was used (Howie et al. 2006). Twenty-four virgin Sprague Dawley rats were housed at 25°C with a 12-h light: 12-h dark cycle. Animals were randomly assigned to one of four diets (Table1) ad libitum throughout pregnancy and lactation (*n *=* *6 per group): (1) standard purified chow diet (CD, 10% kcal from fat); (2) standard purified chow diet with CLA (CLA, 10% kcal from fat, 1% fat from CLA; Stepan Lipid Nutrition, NJ); (3) high fat (HF, 45% kcal from fat); or (4) high fat with CLA (HFCLA, 45% kcal from fat, 1% fat from CLA; Research Diets, Inc., New Brunswick, NJ; Stepan Lipid Nutrition, NJ). Female rats (110 ± 2 days old) were prefed for 10 days prior to being time-mated using an estrous cycle monitor (EC-40, Fine Science Tools, San Francisco, CA). Day 1 of pregnancy was determined by detection of spermatozoa by vaginal lavage, and dams were individually housed. On postnatal day 2 (P2), litter size was randomly adjusted to eight pups (four male, four female) to ensure standardized nutrition until weaning. Pups not allocated to litters were killed by decapitation. All animals were weaned at P21. As we required fasting tissue and plasma samples pups were not killed directly after weaning. Pups were given 3 days to acclimatize and then killed after overnight fasting. At postnatal day 24 (P24), rats were fasted overnight and killed by sodium pentobarbitone (60 mg/kg; IP) anesthesia followed by decapitation. Blood was collected into EDTA tubes and stored on ice until centrifugation and removal of plasma for analysis. Blood glucose was measured directly at the time of cull using a glucose meter (Optium Xceed; Abbott Laboratories). Offspring tissues were collected at P24. One inch segments of intestine were excised approximately 1 cm from the stomach and immediately snap frozen for later analysis. All animal work was approved by the Animal Ethics Committee and the University of Auckland (Approval R1069).

**Table 1:** Composition of experimental diets

	CD		CLA		HF		HFCLA	
	%gm	%kcal	%gm	%kcal	%gm	%kcal	%gm	%kcal
Macronutrient								
Protein	19	20	19	20	24	20	24	20
Carbohydrate	67	70	67	70	41	35	41	35
Fat	4	10	4	10	24	45	24	45
kcal/g	3.8		3.8		4.7		4.7	
Fat composition								
Lard	20	180	19.55	176	177.5	1598	175.5	1579
Soybean oil	25	225	25	225	25	255	25	255
CLA	0	0	0.45	4	0	0	2	18

### Plasma analysis

Fasting plasma insulin (Catalog #90060, Crystal Chem Inc, IL), IL-1*β*, and TNF*α* concentrations (Quantikine kits; R&D Systems Europe, Abingdon, UK) were measured enzymatically. HOMA-IR was calculated as (fasting glucose × fasting insulin/22.5).

### Gene expression analysis

Predesigned probes and TaqMan™ Universal Mastermix were purchased from Applied Biosystems (ABI, CA; Probe information detailed in Table2). All other reagents were purchased from Sigma Aldrich (Auckland, New Zealand) unless otherwise stated. RNA was extracted from upper gut samples, using TRI-Reagent and stored at −80°C. Single-stranded cDNA was prepared using High-Capacity cDNA Archive Kit (Applied Biosystems;Warrington, UK). mRNA expression was quantified by real-time PCR (RT-PCR) on an ABI 7700 Sequence Detection System (PerkinElmer Applied Biosystems). To control for between-sample variability, mRNA concentrations were normalized to the geometric mean of cyclophilinA (PPIA) and hypoxanthine phosphoribosyltransferase 1 (HPRT1) for each sample by subtracting the Ct of controls from the Ct for the gene of interest producing a ΔCt value. The ΔCt for each treatment sample was compared to the mean ΔCt for control samples using the relative quantification 2-(ΔΔCt) method to determine fold-change (Livak and Schmittgen 2014).

**Table 2:** Predesigned probe information

Probe name	ID
Tas1R1	Rn01516038_m1
Tas1R3	Rn00590759_g1
IL-1*β*	Rn00580432_m1
TNF*α*	Rn01525859_g1
NLRP3	Rn04244620_m1
IL-10	Rn01483988_g1
PYY	Rn01460420_g1
Ghrelin	Rn00572319_m1
Cyclophilin A	Rn00690933_m1
HPRT1	Rn01527840_m1

### Statistical analysis

Statistical analysis was performed using SigmaPlot 12.0 (Systat Software Inc., San Jose, CA). All data were analyzed by two-way factorial ANOVA, with maternal high-fat and maternal CLA supplementation as factors. Holm–Sidak post hoc tests were performed where indicated to detect further differences between groups. Differences between groups were considered significant at *P *<* *0.05. All data are presented as means ± SEM unless otherwise stated.

## Results

### Weanling physiological profile:

Physiological data at P24 are presented in Table3. Male and female offspring from HF-fed mothers were significantly heavier than CD, CLA, and HFCLA offspring at P24. While there was no difference in fasting glucose concentrations, HOMA-IR indices were significantly increased in HF compared to CD, CLA, and HFCLA male and female offspring. Furthermore, CLA and HFCLA offspring had significantly lower HOMA-IR indices compared with CD offspring. While there was no significant difference in fasting plasma TNF*α* concentrations, IL-1*β* concentrations were significantly increased in male but not female offspring from HF compared to all other groups.

**Table 3:** Weanling physiological data

	CD	CLA	HF	HFCLA
MALE				
Weight (g)‡	45.88 ± 2.4	44.33 ± 3.3	56.62 ± 2.7*	48.64 ± 3.2†
Glucose (mmol/L)	5.7 ± 0.4	5.8 ± 0.3	6.0 ± 0.7	5.9 ± 0.7
Insulin (ng/mL)§	0.41 ± 0.12	0.27 ± 0.06*	0.73 ± 0.23	0.19 ± 0.01*
HOMA-IR§	0.11 ± 0.02	0.05 ± 0.01*	0.20 ± 0.05*	0.05 ± 0.003*†
IL-1*β* (*ρ*g/mL)‡¶	24.3 ± 2.1	27.98 ± 1.3	33.77 ± 1.8*	29.6 ± 1.8
TNF*α* (*ρ*g/mL)	8.3 ± 1.5	11.8 ± 2.5	11.4 ± 2.4	10.1 ± 2.1
FEMALE				
Weight (g)‡	44.4 ± 1.1	45.6 ± 4.8	58.0 ± 5.0*	47.6 ± 2.8†
Glucose (mmol/L)	5.2 ± 0.1	5.4 ± 0.5	5.3 ± 0.5	4.8 ± 0.3
Insulin (ng/mL)§¶	0.51 ± 0.19	0.54 ± 0.1	1.30 ± 0.38*	0.29 ± 0.11§
HOMA-IR	0.12 ± 0.03	0.14 ± 0.04	0.30 ± 0.10	0.06 ± 0.02
IL-1*β* (*ρ*g/mL)	28.01 ± 2.8	29.86 ± 3.2	29.3 ± 4.6	28.2 ± 3.7
TNF*α* (*ρ*g/mL)	14.8 ± 1.9	10.2 ± 1.6	8.6 ± 0.7	8.1 ± 0.9

Data are presented as means ± SEM. Data were analyzed by two-way ANOVA with maternal HF diet and maternal CLA supplementation as factors. Holm–Sidak post hoc tests were performed where indicated to detect further differences between groups.

* *P* < 0.05 with respect to CD

† *P* < 0.05 with respect to HF

‡ *P* < 0.05 indicates an overall HF effect

§ *P* < 0.05 indicates an overall CLA effect

¶ *P*<0.05 indicates an interaction (HF^*^CLA). HOMA-IR was calculated as: fasting glucose × fasting insulin/22.5.

### Effect of maternal diet on offspring taste receptor expression

**Figure 1:**
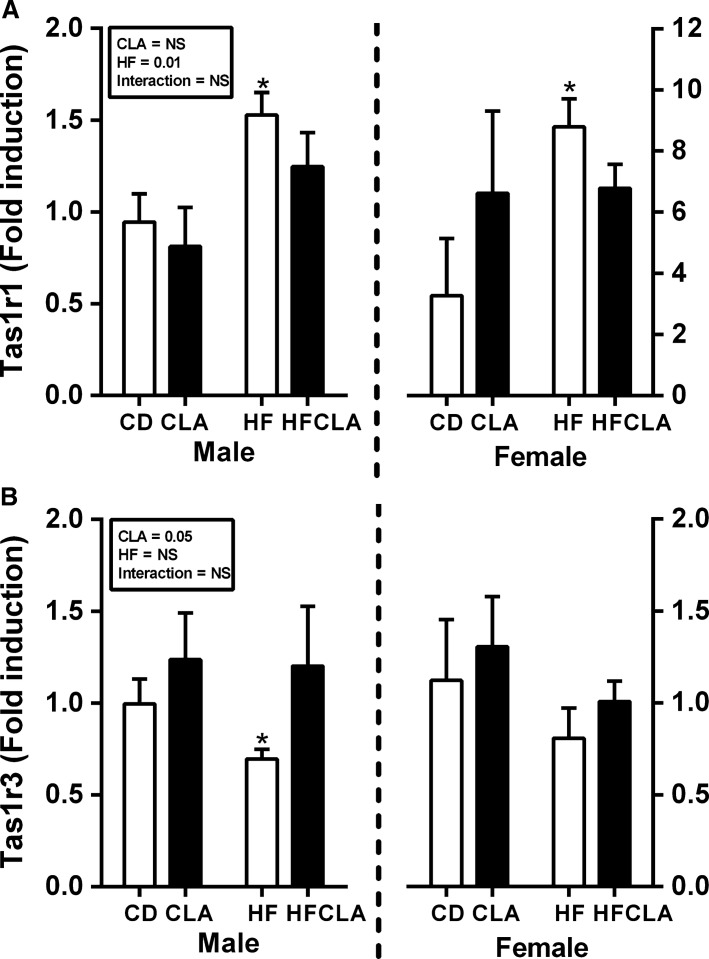
Gut taste receptor expression in offspring at P24. Gut mRNA expression of (A) Tas1R1 and (B) Tas1R3. Data are presented as mean ± SEM. *P <* *0.05 with respect to CD; *n* = 5–6 males; *n* = 5–6 females from independent litters.

Tas1R1 was increased in both male and female offspring from HFD-fed compared to CD but not CLA or HFCLA mothers. Tas1R3 expression was reduced in male but not female offspring of HFD-fed dams compared to all other groups. Furthermore, there was an overall CLA effect in increasing Tas1R3 expression in male offspring (Fig.1). Tas1R2 could not be detected in any of the gut samples examined. CD36, a receptor involved in fat sensing was assessed, however, there were no differences between groups (data not shown).

### Maternal diet alters gut hormone gene expression in offspring

**Figure 2:**
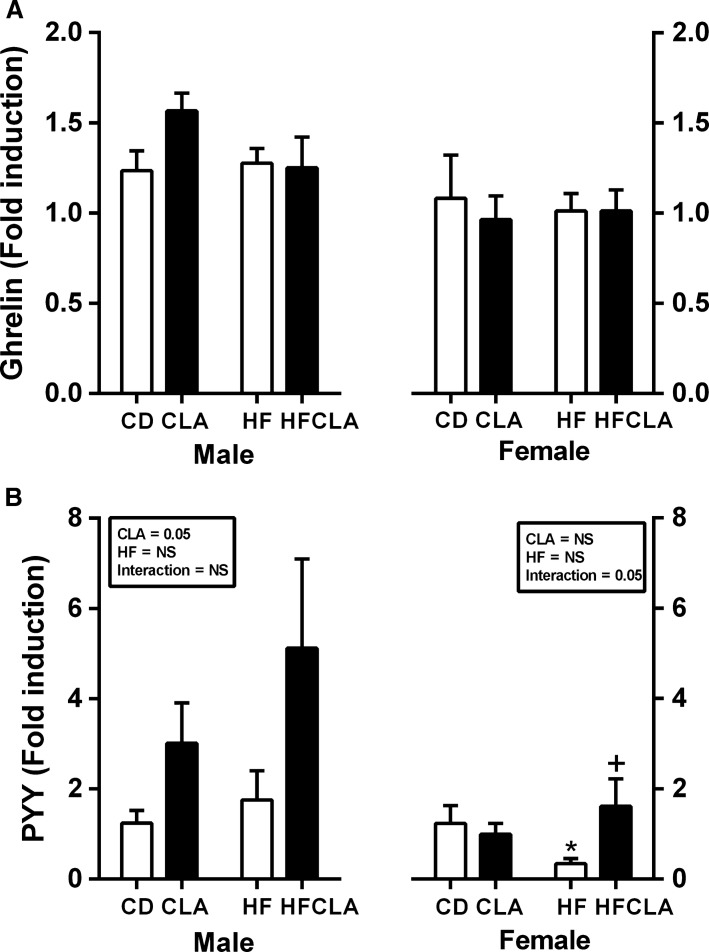
Gut hormone gene expression in offspring at P24. Gut mRNA expression of (A) ghrelin and (B) PYY. Data are presented as mean ± SEM. *P <* *0.05 with respect to CD; ^+^*P* < 0.05 with respect to HF; *n* = 5–6 males; *n* = 5–6 females from independent litters.

There was no significant difference in ghrelin gene expression between groups (Fig.2A). Increased PYY gene expression in CLA and HFCLA contributed to an overall CLA effect in male offspring. However, in female offspring of HF-fed mothers there was a decrease in PYY expression which was reversed as a result of maternal CLA supplementation (Fig.2B).

### Maternal diet alters inflammatory gene expression in male but not female offspring:

**Figure 3:**
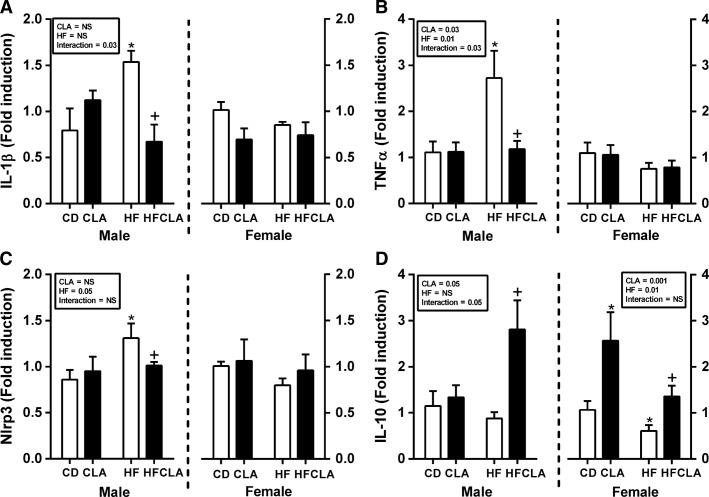
Expression of gut inflammatory markers in offspring at P24. Gut mRNA expression of (A) IL-1*β*, (B) TNF*α*, (C) NLRP3 and (D) IL-10. Data are presented as mean ± SEM. *P < 0.05 with respect to CD; ^+^*P* < 0.05 with respect to HF; *n* = 5–6 males; *n* = 5–6 females from independent litters.

TNF*α*, IL-1*β*, and NLRP3, a component of the IL-1*β* processing machinery, were increased in male but not female offspring of HF-fed compared to CD, CLA, and HFCLA mothers. CLA supplementation in HF-fed mothers significantly ameliorated TNF*α*, IL-1*β*, and NLRP3 gene expression (Fig.3A–C). IL-10 expression was decreased in female offspring of HF-fed mothers compared to CD, CLA, and HFCLA offspring. Furthermore, there was an increase in CLA compared to CD female offspring. In male, HFCLA offspring IL-10 expression was increased compared to all other groups (Fig.3D).

## Discussion

There is clear evidence that dietary intakes during pregnancy have a significant impact on maternal health and long-term obesity and cardiometabolic risk in the offspring. Several biological systems, such as the pancreas, adipose tissue, and liver, have been heavily implicated in this process, with evidence of alterations in cell number, differentiation potential, and organogenesis following exposure to adverse prenatal environments. However, on a mechanistic basis, there is a paucity of data on the role of the gut in mediating of developmental programing-induced pathophysiologies despite significant evidence that gut function strongly influences obesity and metabolic dysfunction. Imbalances in gut microbiome composition and alterations in gut structure, nutrient transport, and hormonal regulation are cited as potential mechanisms which initiate the onset of metabolic disease (Ma et al. 2006). Thus, while the link between gut biology and metabolic dysfunction has been comprehensively established in the broad setting of obesity and related metabolic disorders, there is limited evidence in regard to the role of maternal programing on gut biology in offspring. There is human and experimental evidence, albeit limited, for an effect of maternal diet on setting of taste preferences in offspring and a preference for fatty foods (Bellinger et al. 2012; Lussana et al. 2014), although the potential for this to be mediated via changes in gut taste receptor sensing has not been explored. The current study therefore examined gut gene expression of taste receptors, inflammatory markers, and gut hormones in offspring from HF-fed mothers supplemented with or without the anti-inflammatory lipid CLA.

Similar to other published work, we demonstrate that offspring of HF-fed mothers are heavier and have increased insulin concentrations and HOMA-IR indices at the end of the weaning period (Reynolds et al. 2004). We further demonstrate that maternal supplementation with an anti-inflammatory lipid, CLA, resulted in a reversal of the decreased birth weights observed in offspring (both male and female) from HF mothers, this subsequently prevented preweaning catch-up growth and reversed glucose intolerance and increased weaning weights in both male and female offspring (Segovia et al. 2015). We have also shown that between puberty and adulthood male offspring from HF mothers remain heavier and have poorer glucose control by P150, while in females there was no difference in weight or glucose control. This may be influenced by the effects of estrogen in females postpuberty. CLA mediates a range of metabolic processes including adipogenesis, inflammation, and lipid metabolism (Moloney et al. 2014). There is also clear evidence regarding the beneficial role of CLA in gut disorders such as inflammatory bowel disease and colon cancer (Bassaganya-Riera et al. 2010). In addition to determining the role of maternal HF diet on developmental programing in the gut, we examined the impact of maternal CLA supplementation on offspring (P24) gut gene expression.

The gastrointestinal system represents the primary interface between the body and ingested food and as such regulates the absorption and digestion of nutrients. Similar to the tongue, the gut contains “tastebuds” composed of receptors which bind to sweet, sour, fatty, bitter, and salty compounds (Laffitte et al. 2007). Upon activation, these taste receptors promote secretion of bioactive gut peptides, PYY, GLP-1, and ghrelin, which in turn govern appetite regulation, digestive function, and glucose release from the gut (Jang et al. 2013; Kokrashvili et al. 2011). Dysregulation of this gut sensory system has now been recognized as a potential mechanism for obesity and metabolic dysfunction (Nguyen et al. 2005). The current study demonstrates for the first time that offspring from HF-fed mothers have altered expression of gut taste receptors Tas1R1 and Tas1R3 at the time of weaning. Interestingly, there is a partial normalization of these changes in response to maternal CLA supplementation. Tas1R1, which is traditionally viewed as a broad sensor of amino acid concentrations, is significantly increased in offspring, both male and female, from HF-fed mothers. Several studies have associated elevated Tas1R1 with metabolic disease. Evidence from Wauson et al. indicates that depletion of this receptor in mice reduces responsiveness to both amino acids and sweeteners via reduced activation of the nutrient sensing mTORC1 pathway and a subsequent increase in autophagy. We also demonstrate dysregulated expression of Tas1R3 sweet taste receptor in male offspring from HF-fed mothers with a normalization following maternal CLA supplementation. Dysregulation of this gut sensory system has now been recognized as a potential mechanism for obesity and metabolic dysfunction (Sprous and Palmer 2014). Indeed emerging evidence suggests that aberrant activation of taste receptors in the upper gut of obese individuals increases expression of the glucose transporters, SLGT1 and GLUT1, promoting systemic insulin resistance (Nguyen et al. 2005). Evidence from Roux-en-Y gastric bypass surgery demonstrates that removal of the region of the gut which contains taste receptors can reverse obesity-induced metabolic dysfunction (Mokadem et al. 2007). It should be noted that while data is lacking, T1R1 and T1R3 are known to form heterodimers which recognize umami compounds, therefore we cannot discount the possibility of functional heterodimer formation. Therefore, findings from the current study may indicate that reprograming of taste receptors in the gut by maternal CLA supplementation may represent a viable early-life treatment option for adult onset obesity and diabetes.

As detailed above, activation of taste receptors promote release of key gut hormones which influence glucose homoeostasis. While there was no difference in gene expression of the “hunger hormone” ghrelin in offspring upper gut, PYY was significantly changed in a sex-specific manner. PYY is a peptide hormone which reduces appetite by slowing gastric emptying and increases the efficiency of digestion. In humans, both nonpregnant and pregnant, there is a negative association between PYY concentrations and satiety in obese adults (Guo et al. 2014; le Roux et al. 2007; Sodowski et al. 2011). In the current study, there is a significant decrease in PYY gene expression in female but not male offspring of HF-fed mothers, which may in part contribute to the observed obese phenotype at weaning. Consistent with evidence that CLA stimulates PYY secretion in a murine cell line (STC-1) (Hand et al. 2012), CLA promotes increased PYY gene expression in both male and female offspring from CLA supplemented mothers. As animals in this study were directly exposed to CLA via breast milk it remains unclear whether the stimulation of PYY gene expression is due to direct exposure or programed effects. In addition, it should be noted that expression was only measured in the duodenum which only represents approximately 10% of PYY expression in the gut. Irrespective of this, promotion of PYY secretion may act to prevent obesity in these offspring early in life and therefore promote a healthier metabolic phenotype during adulthood.

Given the association between metabolic dysfunction and low-grade inflammation, we examined the inflammatory profile in the offspring gut at weaning. While inflammatory cytokines are essential to gut homeostasis in low concentrations, excessive production can result in intestinal permeability and even chronic conditions such as inflammatory bowel disease (Mowat and Bain 2011; Bain and Mowat 2011). TNF*α* and IL-1*β* are potent proinflammatory cytokines which are heavily associated with gut permeability, metabolic dysfunction, and chronic autoimmune conditions (McGillicuddy et al. 2001; Coccia et al. 2004; Gersemann et al. 2012). TNF*α* is secreted in an active form, however, activation of IL-1*β* requires two distinct signals, expression of pro-IL-1*β* and inflammasome-mediated cleavage of the propeptide by caspase-1 to form mature IL-1*β* (Stienstra et al. 2008). NLRP3 inflammasome regulation of IL-1*β* activation is associated with both metabolic inflammation and gut dysregulation (Vandanmagsar et al. 2014; Zaki et al. 2010). The current study demonstrates increased expression of TNF*α*, IL-1*β*, and NLRP3, in male but not female, offspring from HF-fed mothers. Interestingly, maternal CLA supplementation reversed TNF*α*, IL-1*β*, and NLRP3 expression profiles. There is significant evidence that supplementation with CLA ameliorates inflammation in a range of tissue types (Moloney et al. 2008; Bassaganya-Riera and Hontecillas 2014; Draper et al. 2015). However, it is unclear whether this is a direct effect of CLA in offspring gut via consumption of milk or an indirect effect of programing. Further work is required to determine if these effects persist to adulthood.

As it is clear that maternal HF feeding instigates a proinflammatory response in the gut of offspring at weaning, we examined expression of the immuno-modulatory cytokine IL-10. This cytokine plays a key role in gut homeostasis. Disruption of IL-10 signaling is a key determinant in chronic inflammatory disease in the gut and has been explicitly demonstrated in IL-10 knockout mice who spontaneously develop severe colitis (Kuhn et al. 2009). Furthermore, several studies have indicated a protective role for IL-10 in obesity-induced metabolic dysregulation, with evidence that concentrations are reduced in patients with insulin resistance and type 2 diabetes (van Exel et al. 2007; Lumeng et al. 1993). The current study demonstrated a significant decrease in IL-10 gut gene expression in female but not male offspring from HF-fed mothers. This sex-specific effect on IL-10 may indicate that gut dysfunction may arise via differential signaling pathways in this model of developmental programing. Interestingly, maternal supplementation with CLA increases gut IL-10 gene expression in both male and female offspring. The anti-inflammatory effects may, at least in part, explain the reduction in proinflammatory cytokines in male offspring.

This study has confirmed previous work demonstrating that a maternal HF diet during gestation and lactation promotes increased weight gain during the weaning period accompanied by evidence of metabolic dysregulation. While it is clear that maternal diet plays a major role in the predisposition to obesity and metabolic disease, the mechanisms are not comprehensively understood, particularly in relation to gut biology. This study demonstrates that the gut may play a key part in early-life glucose dysregulation which precedes overt metabolic disease. HF diet exposure during early life influences expression of key mediators of gut glucose regulation, including taste receptors and incretins, which may promote increased indices of insulin resistance in these offspring. Furthermore, we demonstrate an increase in proinflammatory cytokine expression which may advocate a role for maternal diet in “leaky gut” syndrome and inflammatory bowel diseases in later life. We have no direct evidence to suggest that the gut sensory system is dysregulated, however, this may be a possibility for the lack of correlation between Tas1R1 and the gut hormone PYY. It should be noted that PYY is also produced in other regions of the gastrointestinal tract, therefore, it may be possible that expression in these regions may more accurately reflect the patterns observed in Tas1R1 expression. Furthermore, given the role of cytokines in normal gut function, it is likely that dysregulated inflammatory processes in the gut could influence the gut sensory system in this model, however, further experiments would be required to confirm this. There is significant evidence that intervention early in life is the most effective strategy for preventing developmental programing of metabolic dysfunction. This study establishes that the anti-inflammatory lipid, CLA, is effective in reversing the detrimental effects of maternal high-fat during pregnancy and lactation. Given the growing rates of obesity worldwide this research is important in terms of gaining understanding of the mechanisms involved in programing risk of metabolic dysfunction thereby providing a starting point for development of therapeutic strategies.
